# Supplementary material for: CTLA-4 expressing innate lymphoid cells modulate mucosal homeostasis in a microbiota dependent manner
Source: Nat Commun. 2024 Nov 4;15:9520. doi: 10.1038/s41467-024-51719-6 (PMC11535242; doi:10.1038/s41467-024-51719-6)
Supplement: Supplementary file 1 — Supplementary Information [file 41467_2024_51719_MOESM1_ESM.pdf]

Supplementary Information for “CTLA-4 expressing innate lymphoid cells modulate mucosal homeostasis in a microbiota dependent manner”

By: Jonathan W. Lo et al.

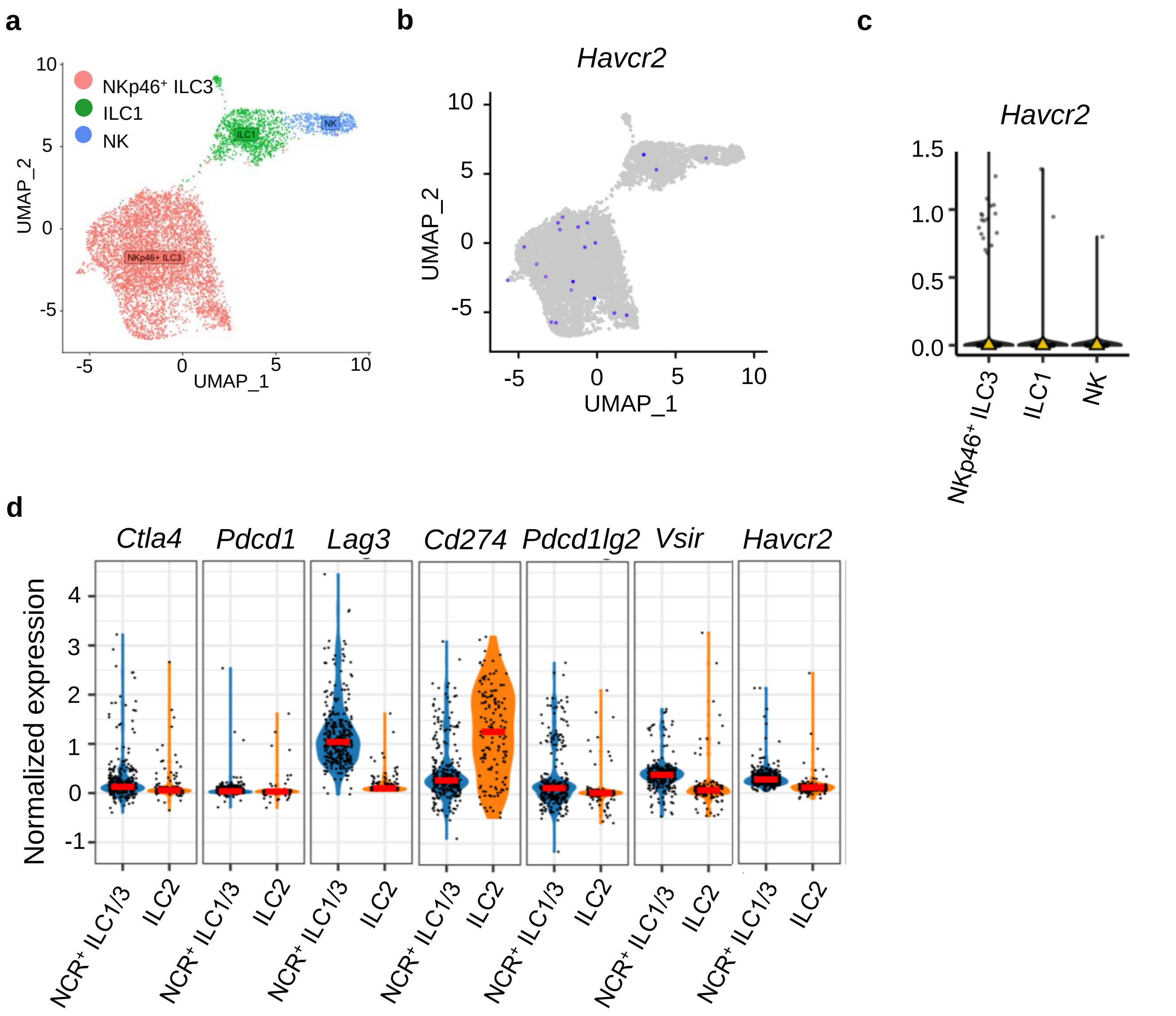

Supplementary Figure 1. The immune checkpoint transcriptional landscape across murine intestinal ILC clusters

(a) UMAP plot showing the three populations of NKp46+ sorted cells previously identified by Krzywinska et al. **(b)** UMAP plots and **(c)** violin plots showing the expression of Havcr2 across the three subsets of ILC identified by Krzywinska et al. (median shown by the yellow triangle). **(d)** Violin plots showing the expression of canonical immune checkpoints across the two ILC subsets identified in CD45^{+} sorted cells from healthy wild-type BALB/c mice.

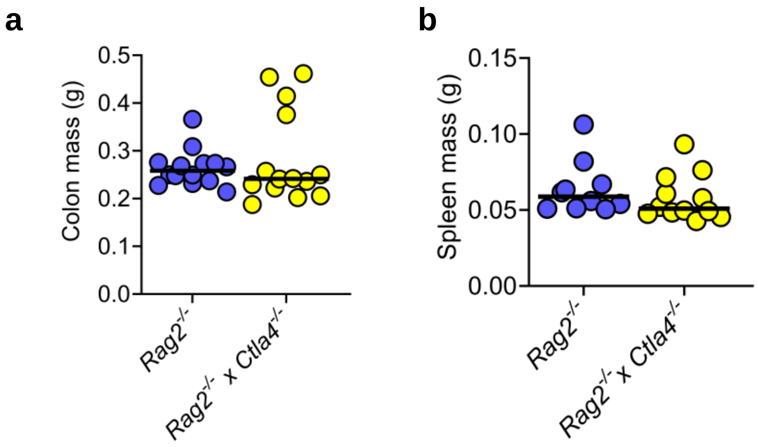

Supplementary Figure 2. Lack of spontaneous inflammatory disease in *Rag2$^{-/-}$ x Ctla4$^{-/-}$* mice

(a) Summary statistics showing colon and **(b)** spleen mass between *Rag2$^{-/-}$* mice (n=14 for colon, n=10 for spleen) and *Rag2$^{-/-}$ Ctla4$^{-/-}$* mice (n=14 for colon, n=12 for spleen).

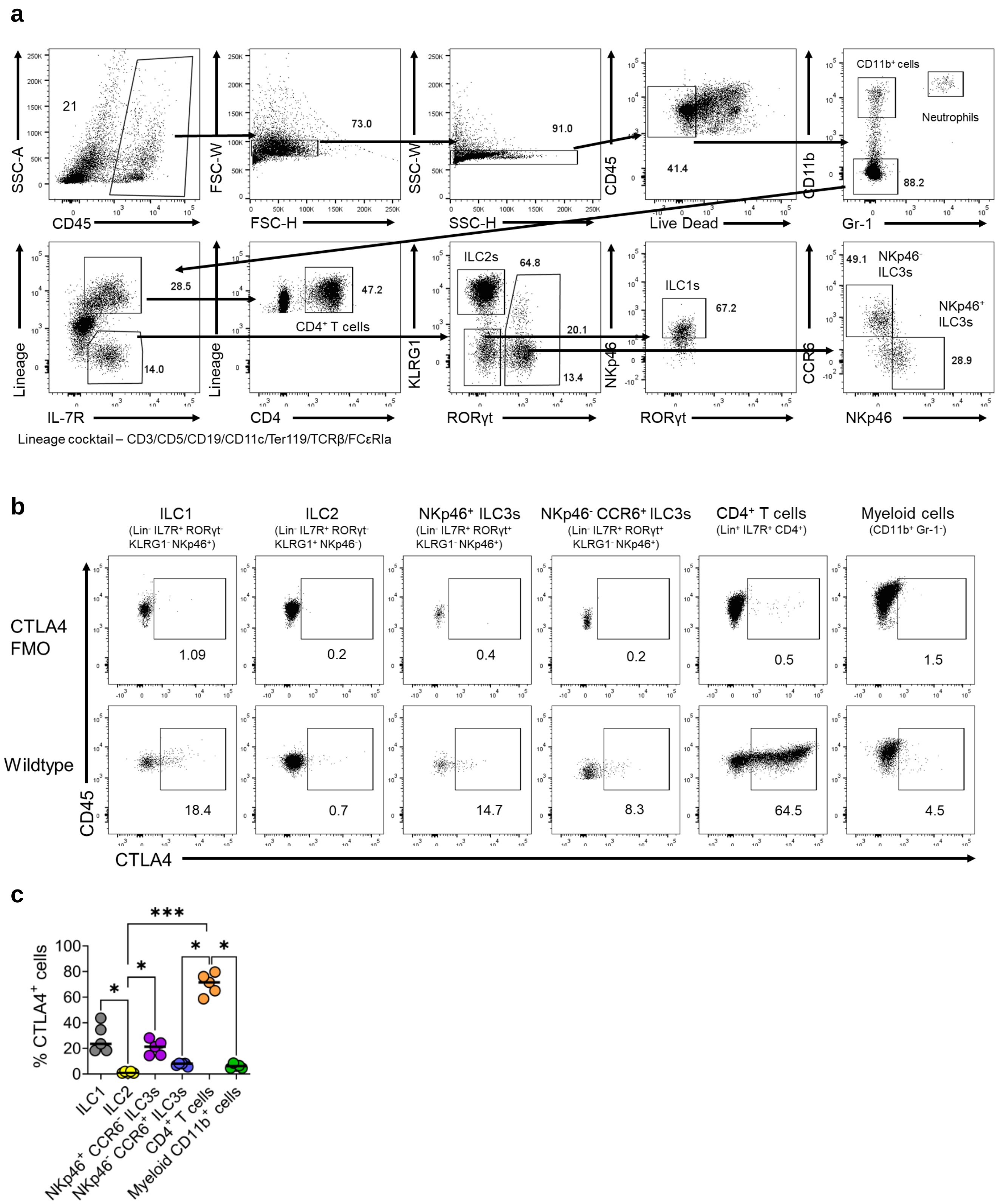

Supplementary Figure 3. Comparison of CTLA-4 expression between the ILC subsets and other immune cells

(a) Gating strategy used to identify ILCs, T cells and CD11b myeloid cells from the colonic lamina propria. **(b)** Representative dot plot and **(c)** summary dot plot showing the comparison of the percentage of CTLA-4 expressing cells across different immune cells found in the colonic lamina propria (n=5) * P<0.05 ** P<0.01 ** P<0.001 using a Kruskall-Wallis Test.

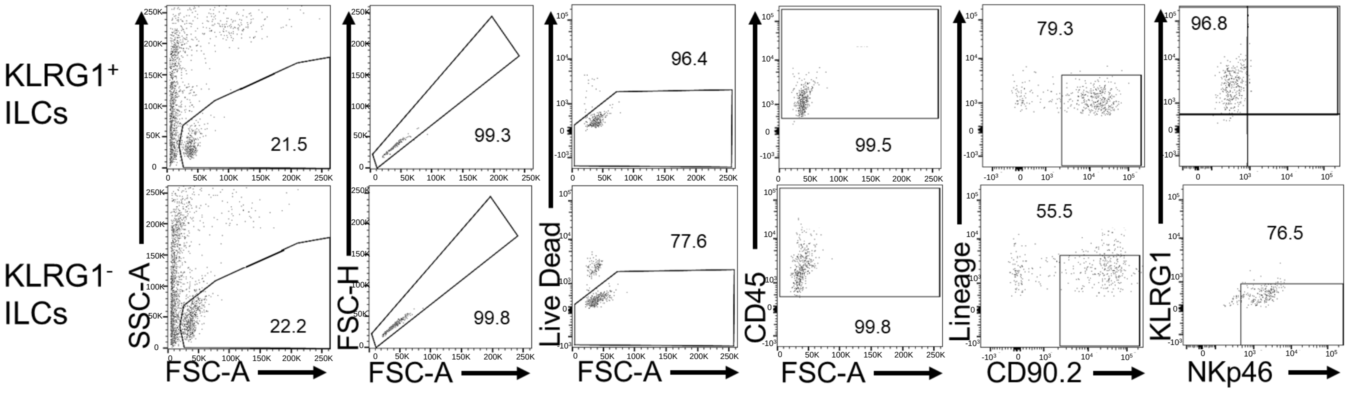

Supplementary Figure 4. Gating strategy for *in vitro* cultured murine ILC subsets
Gating strategy used in wild-type mice to identify KLRG1⁺ and KLRG1⁻ ILCs.

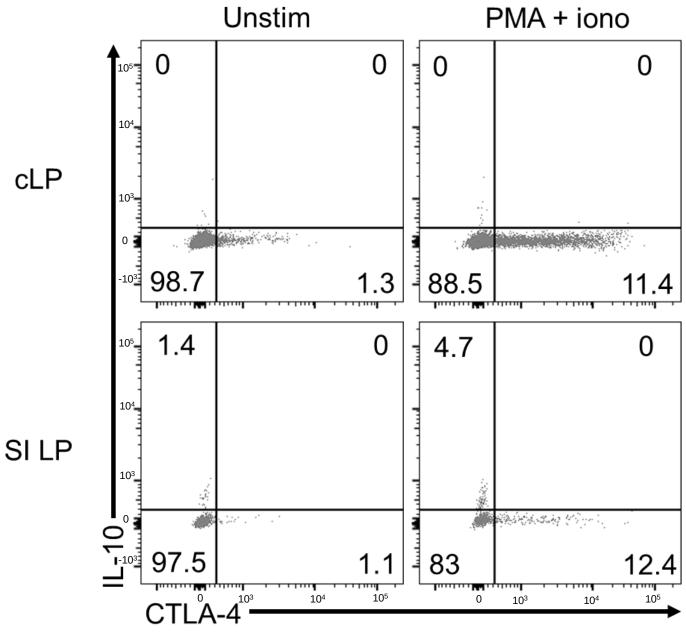

Supplementary Figure 5. CTLA-4 expressing ILCs do not produce IL-10
Representative flow cytometry plot showing IL-10 production from CTLA-4^{+} ILCs after 4 hours of PMA and ionomycin stimulation (n=3)

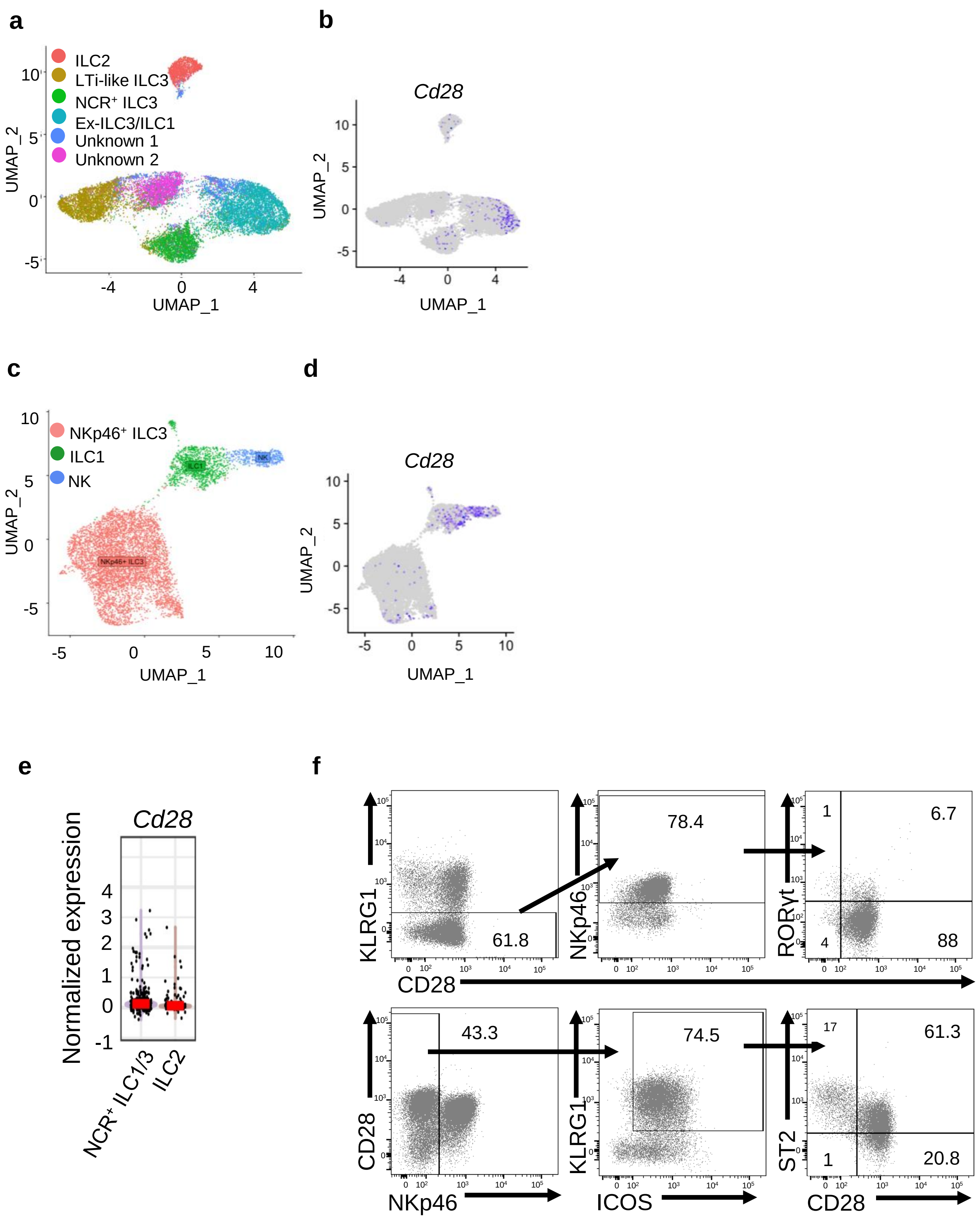

Supplementary Figure 6. CD28 is expressed across murine ILC subsets using both single cell RNA-seq and flow cytometry

(a) UMAP plot showing the six ILC populations previously identified from CD45$^+$ Lin$^-$ CD127$^+$ sorted cells by Fiancette et al. **(b)** UMAP plot showing the expression of *Cd28* across the six identified ILC subsets. **(c)** UMAP plot showing the three populations of Nkp46$^+$ sorted cells previously identified by Krzywinska et al. **(d)** UMAP plot showing the expression of *Cd28* across the three identified ILC populations. **(e)** Violin plot showing the expression of *Cd28* across the two identified ILC populations identified from Lo et al.. **(f)** cLP leukocytes were isolated from BALB/c mice for flow cytometry analysis. Representative flow plots showing CD28 expression in KLRG1$^-$ NKp46$^+$ ILC1 and ILC3 and NKp46$^-$ ICOS$^+$ KLRG1$^+$ ST2$^{-/+}$ ILC2 are demonstrated.

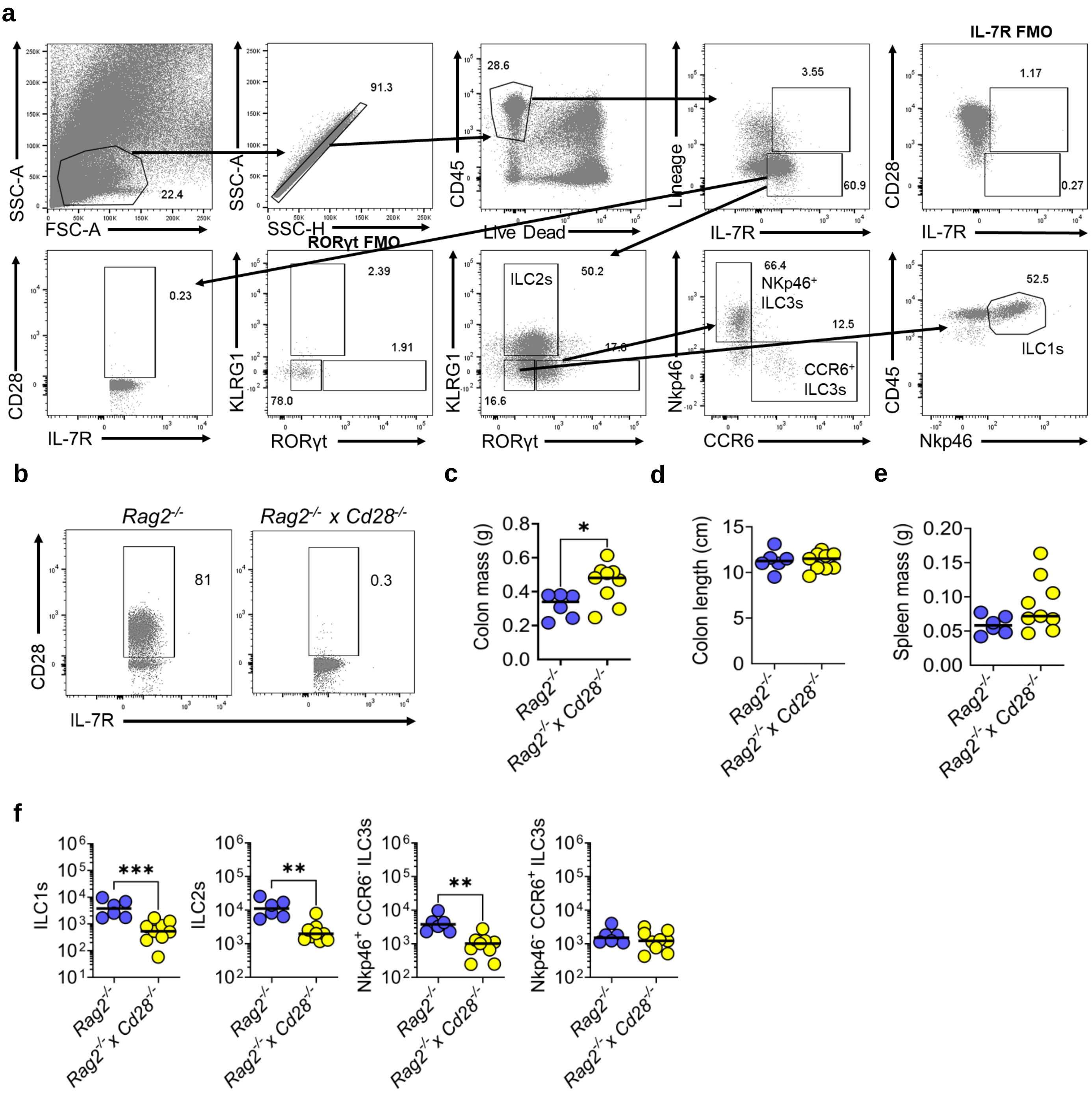

Supplementary Figure 7. CD28 can modulate ILC numbers in the colon

(a) Gating strategy used to identify ILCs in *Rag$^{-/-}$ x Cd28$^{-/-}$* **(b)** Representative flow plot showing loss of CD28 in *Rag$^{-/-}$ x Cd28$^{-/-}$* **(c)** Summary dot plot showing colon mass, **(d)** colon length and **(e)** spleen mass between *Rag$^{-/-}$* (n=6) and *Rag$^{-/-}$ x Cd28$^{-/-}$* (n=9) **(f)** Summary dot plot absolute cell numbers in the 4 ILC populations identified in the colonic lamina propria between *Rag$^{-/-}$* (n=6) and *Rag$^{-/-}$ x Cd28$^{-/-}$* (n=9). * P<0.05 ** P<0.01 *** P<0.001 Mann Whitney U Test for d-f.

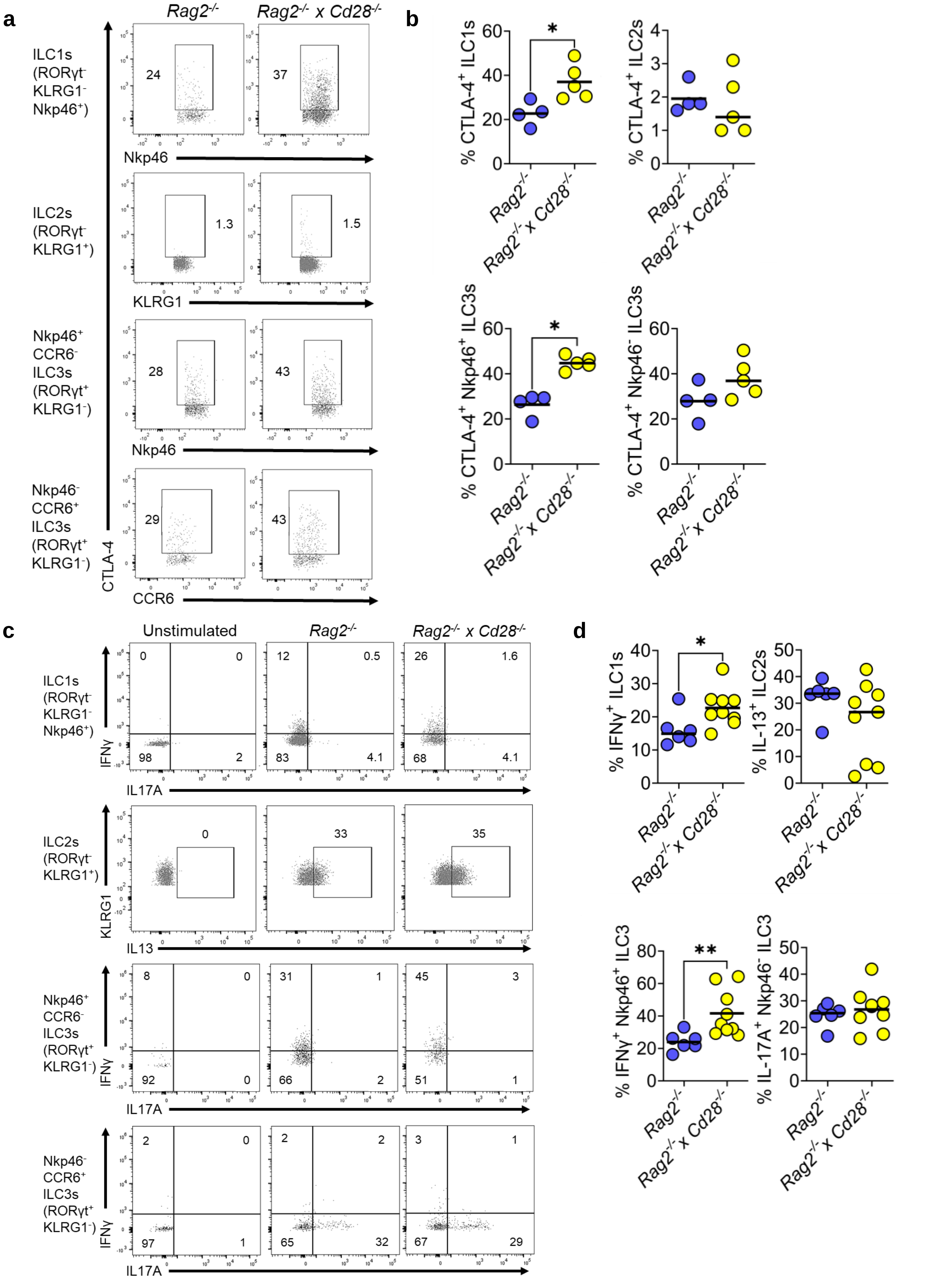

Supplementary Figure 8. CD28 can modulate CTLA-4 expression and cytokine production
(a) Representative flow plots and **(b)** summary dot plot of CTLA-4⁺ ILC in the 4 populations identified between *Rag*$^{-/-}$ (n=4) and *Rag*$^{-/-}$ *x Cd28*$^{-/-}$ (n=5) after PMA and ionomycin stimulation **(c)** Representative flow plots and **(d)** Summary dot plot showing changins in cytokines in the 4 identified ILC subsets between *Rag*$^{-/-}$ (n=6) and *Rag*$^{-/-}$ *x Cd28*$^{-/-}$ (n=9). * P<0.05 ** P<0.01 Mann Whitney U Test for b and d.

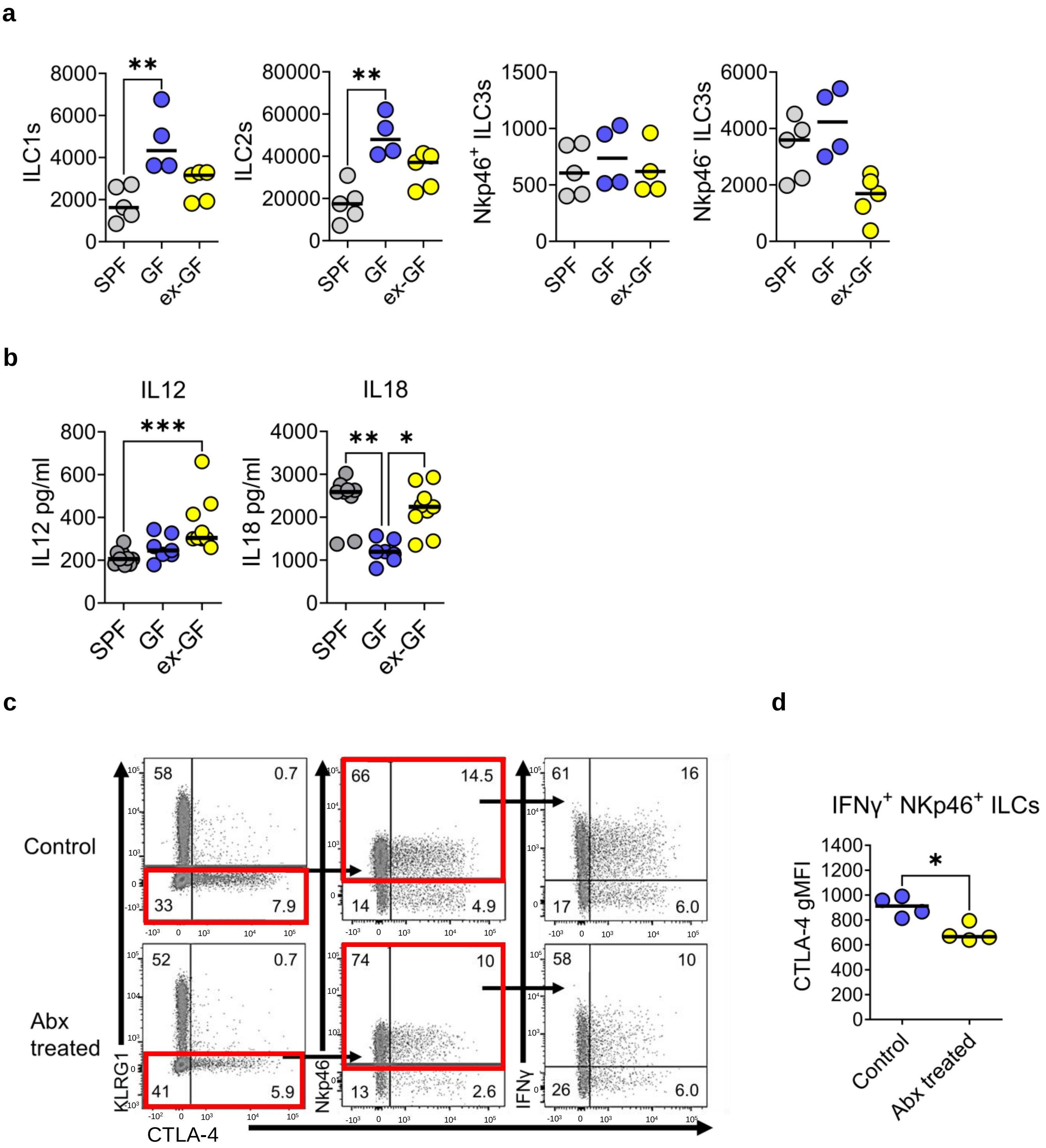

Supplementary Figure 9. CTLA-4 expression is affected by the composition of the microbiota
(a) Summary dot plot of absolute cell numbers within the colonic lamina propria for the four ILC subsets shown from SPF mice (n=5), GF mice (n=4) and ex-GF mice (n=5) **(b)** Summary dot plot of IL12 and IL18 using ELISA from colonic biopsies cultured for 24 hours from SPF mice (n=9), GF mice (n=7) and ex-GF mice (n=9) **(c)** Representative flow plots and **(d)** summary dot plot showing changes in gMFI of CTLA-4 in ILCs between control C57BL/6 wildtype mice (n=4) and C57BL/6 wildtype mice treated with antibiotics (n=4). * P<0.05 ** P<0.01 *** P<0.001 Kruskal-Wallis Test for a and b, Mann Whitney U Test for d.

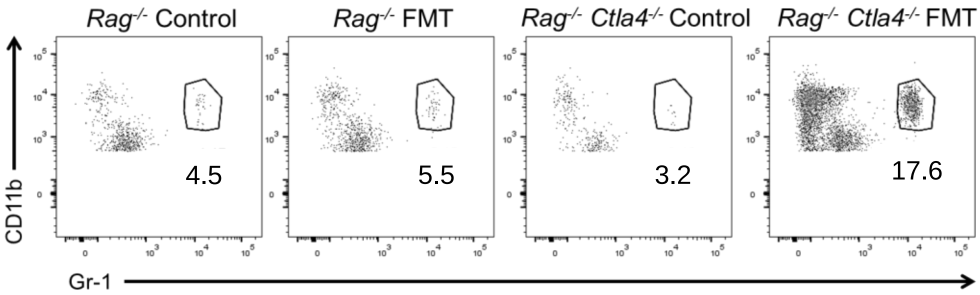

Supplementary Figure 10. FMT causes an increase in neutrophils in *Rag2$^{-/-}$* x *Ctla4$^{-/-}$* mice
Representative flow plots showing CD11b^{+} Gr-1^{+} neutrophils in *Rag2$^{-/-}$ x Ctla4$^{-/-}$* mice.

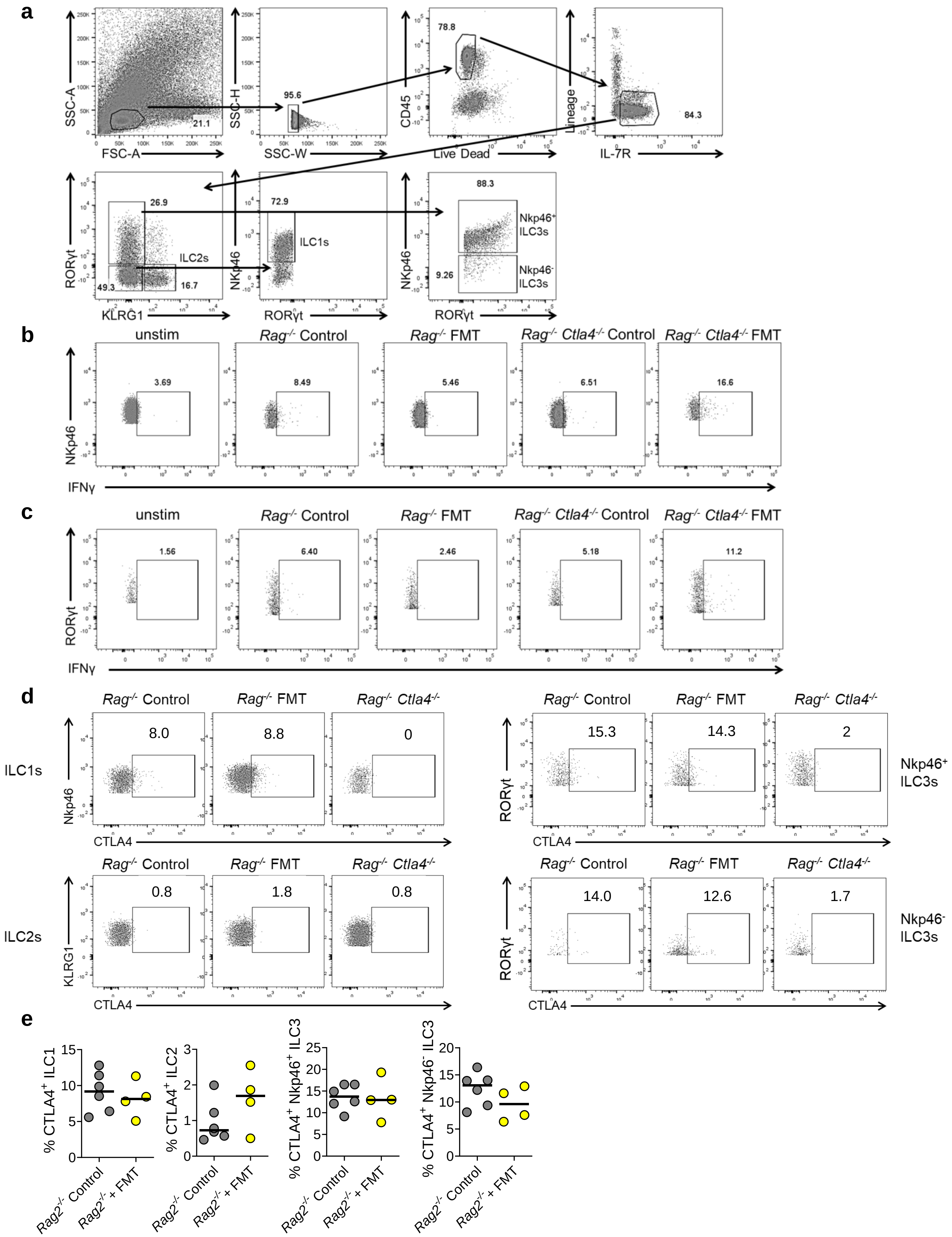

Supplementary Figure 11. FMT causes an increase in IFNγ in *Rag2$^{-/-}$ x Ctla4$^{-/-}$* mice
(a) Gating strategy for identifying the different subsets of ILCs in *Rag2$^{-/-}$ x Ctla4$^{-/-}$* mice. **(b)** Representative flow plots showing IFNγ producing ILC1s and **(c)** Nkp46^{+} ILC3s. **(d)** Representative and **(e)** summary dot plot showing CTLA-4 expression in the 4 ILC subsets identified in *Rag2$^{-/-}$* (n=6) compared to FMT treated *Rag2$^{-/-}$* (n=4).

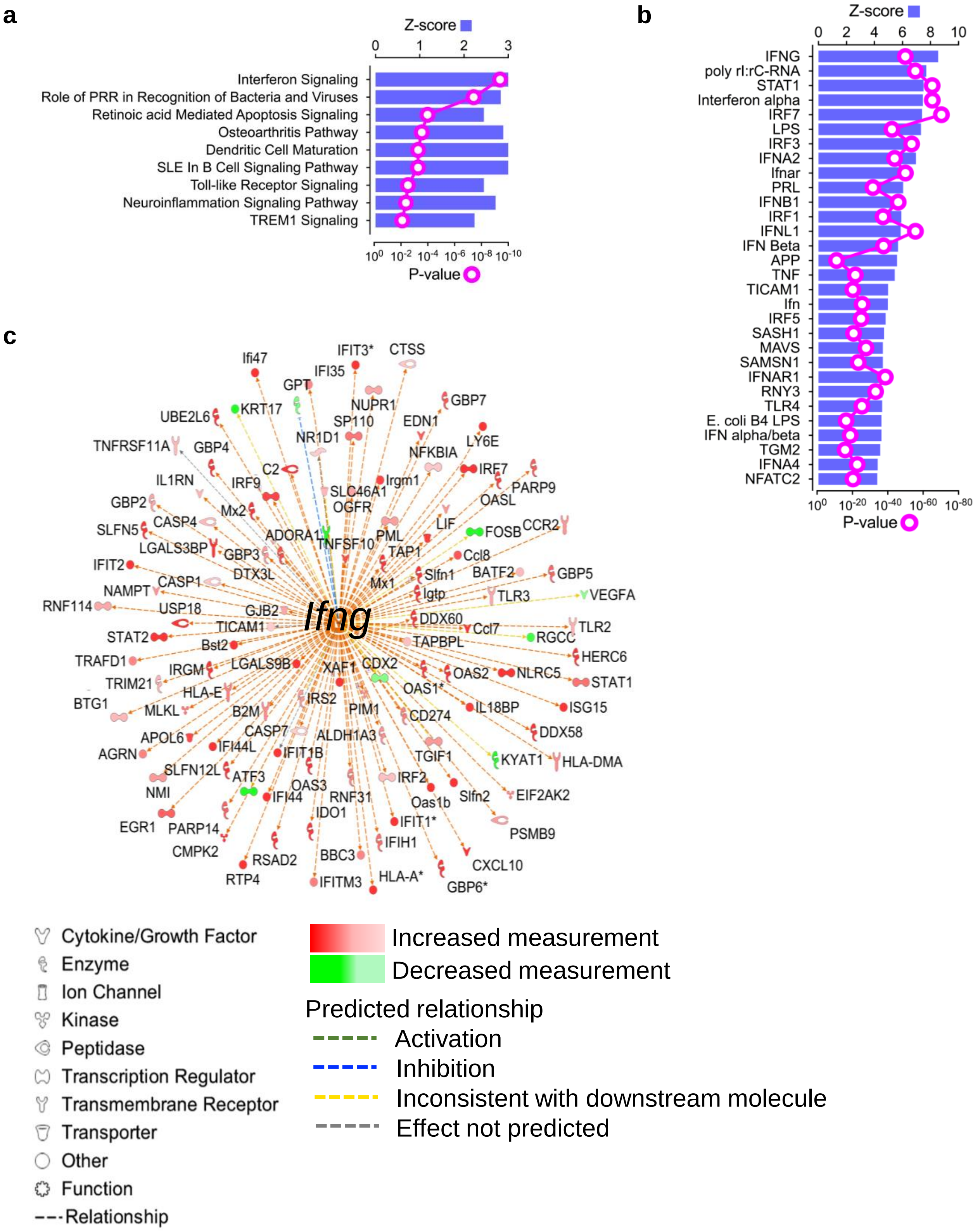

Supplementary Figure 12. Pathway analysis and upstream network analysis identifies enrichment of IFNγ regulated biological pathways in *Rag2$^{-/-}$ x Ctla4$^{-/-}$*

(a) Canonical pathways (Ingenuity Pathway Analysis, IPA) activated in colon segments from *Rag2$^{-/-}$ x Ctla4$^{-/-}$* mice (n=4) compared to *Rag2$^{-/-}$* mice (n=4). **(b)** Upstream regulators predicted to control the gene expression changes observed in *Rag2$^{-/-}$ x Ctla4$^{-/-}$* mice (n=4) compared to *Rag2$^{-/-}$* mice (n=4). **(c)** Causal network analysis of the top predicted upstream regulator (*Ifng*) observed in the colon of *Rag2$^{-/-}$ x Ctla4$^{-/-}$* mice (n=4) in comparison with *Rag2$^{-/-}$* mice (n=4), using IPA.

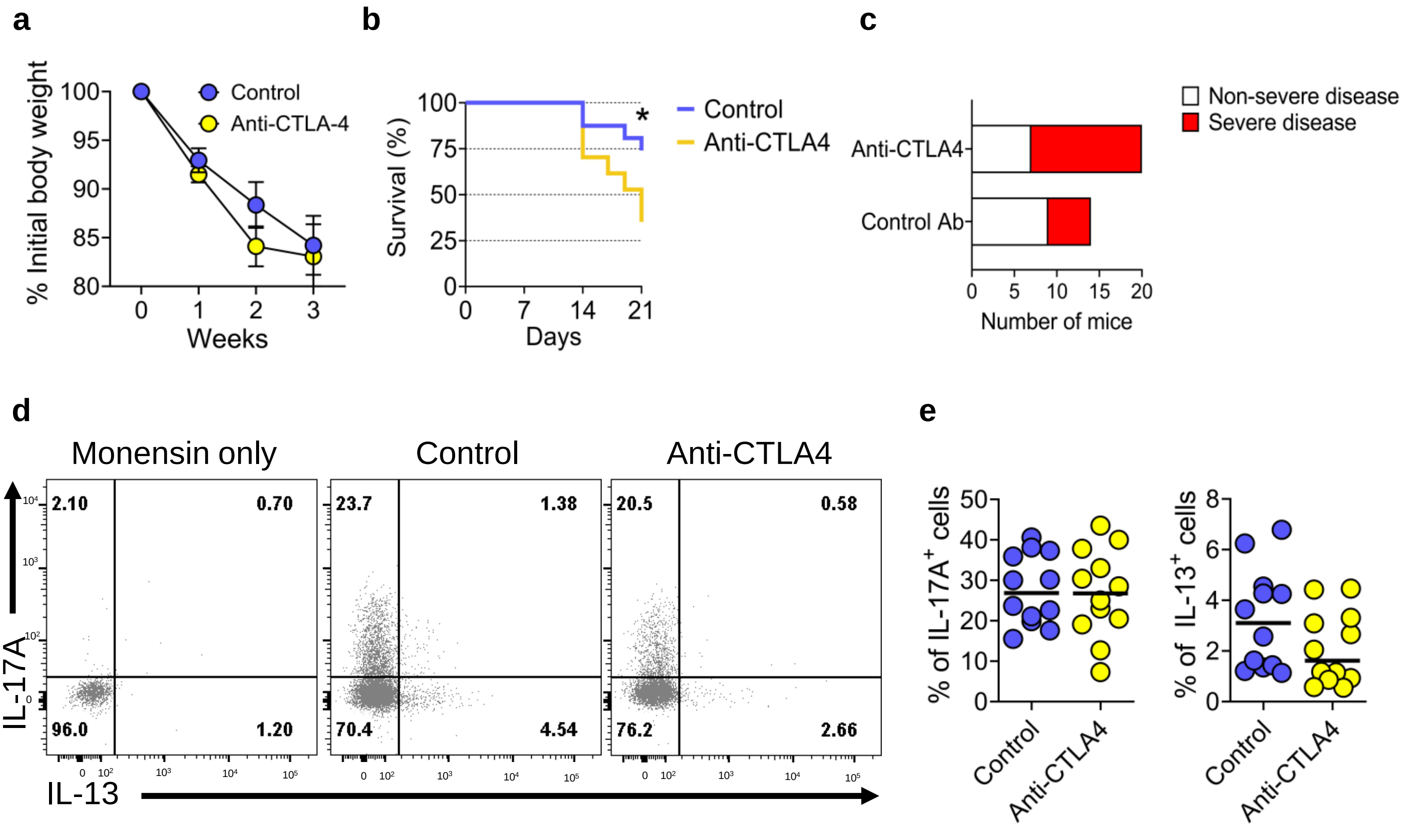

Supplementary Figure 13. TRUC mice suffer worse disease when treated with CTLA-4 blockade
(a) Weight loss, **(b)** survival curve and **(c)** severity index (based on weight loss of <15% initial body weight or having to be culled before the end point) between TRUC untreated mice (control) (n=12) compared to TRUC mice treated with anti-CTLA-4 (n=12) * P<0.05 Logrank test. **(d)** Representative flow plot and **(e)** summary dot plot showing IL-17A and IL-13 producing ILCs between TRUC untreated mice (control) (n=12) compared to TRUC mice treated with anti-CTLA-4 (n=12).

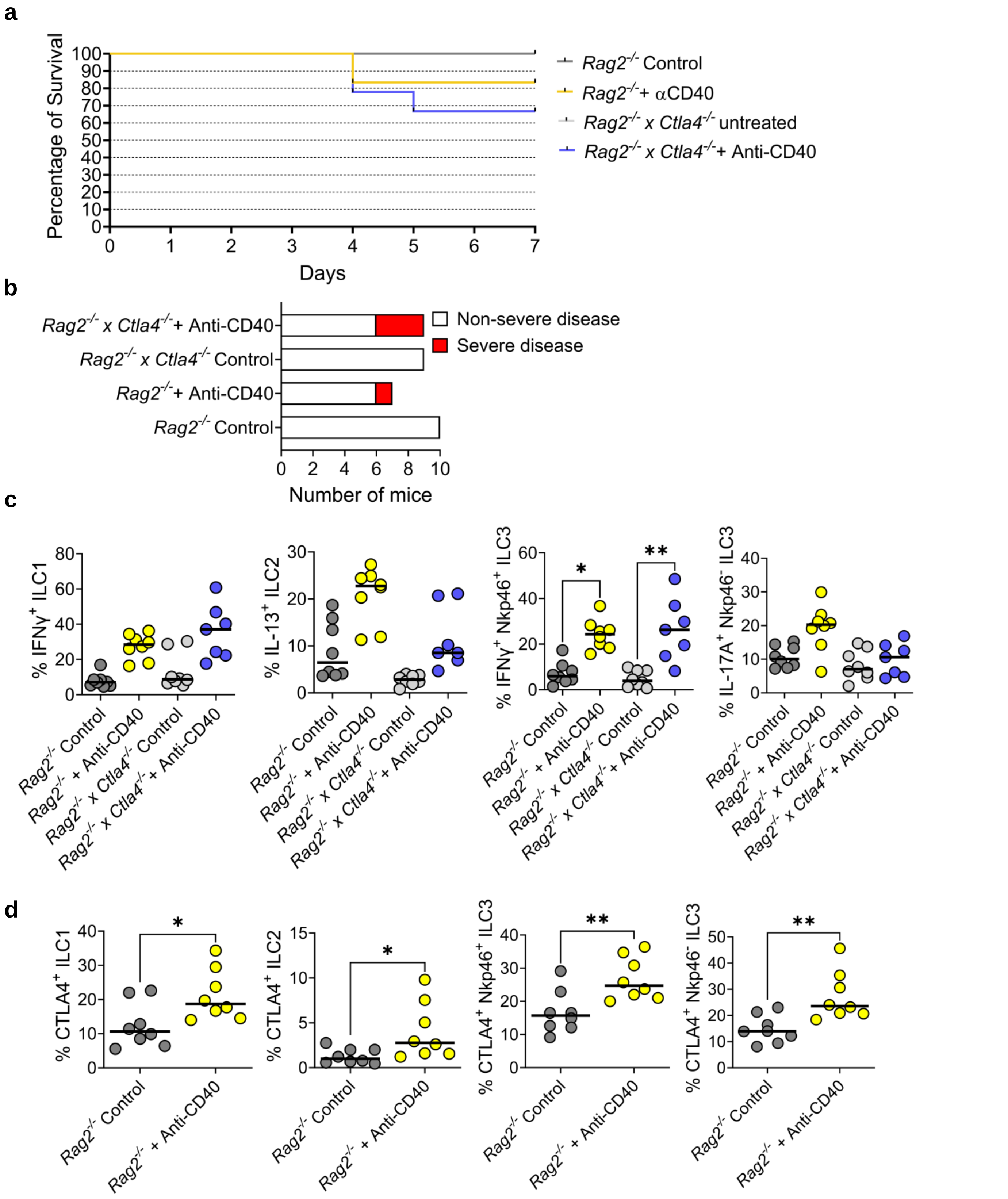

Supplementary Figure 14. *Rag2$^{-/-}$* mice deficient for CTLA-4 suffer worse disease when treated with anti-CD40 **(a)** Survival curve and **(b)** severity index (based on weight loss of <15% initial body weight or having to be culled before the end point) between control untreated BALB/c *Rag2$^{-/-}$* mice (n=8), BALB/c *Rag2$^{-/-}$* mice treated with anti-CD40 (n=8), untreated BALB/c *Rag2$^{-/-}$ x Ctla4$^{-/-}$* mice (n=8) and BALB/c *Rag2$^{-/-}$ x Ctla4$^{-/-}$* mice treated with anti-CD40 (n=7). **(c)** Summary dot plot showing cytokine producing ILCs between control untreated BALB/c *Rag2$^{-/-}$* mice (n=8), BALB/c *Rag2$^{-/-}$* mice treated with anti-CD40 (n=8), untreated BALB/c *Rag2$^{-/-}$ x Ctla4$^{-/-}$* mice (n=8) and BALB/c *Rag2$^{-/-}$ x Ctla4$^{-/-}$* mice treated with anti-CD40 (n=7). (d) Summary dot plot showing CTLA-4 expressing ILCs between between control untreated BALB/c *Rag2$^{-/-}$* mice (n=8), BALB/c *Rag2$^{-/-}$* mice treated with anti-CD40 (n=8). * P<0.05 ** P<0.01 *** P<0.001 2-sided Kruskal-Wallis Test for c and Mann Whitney U Test for d.

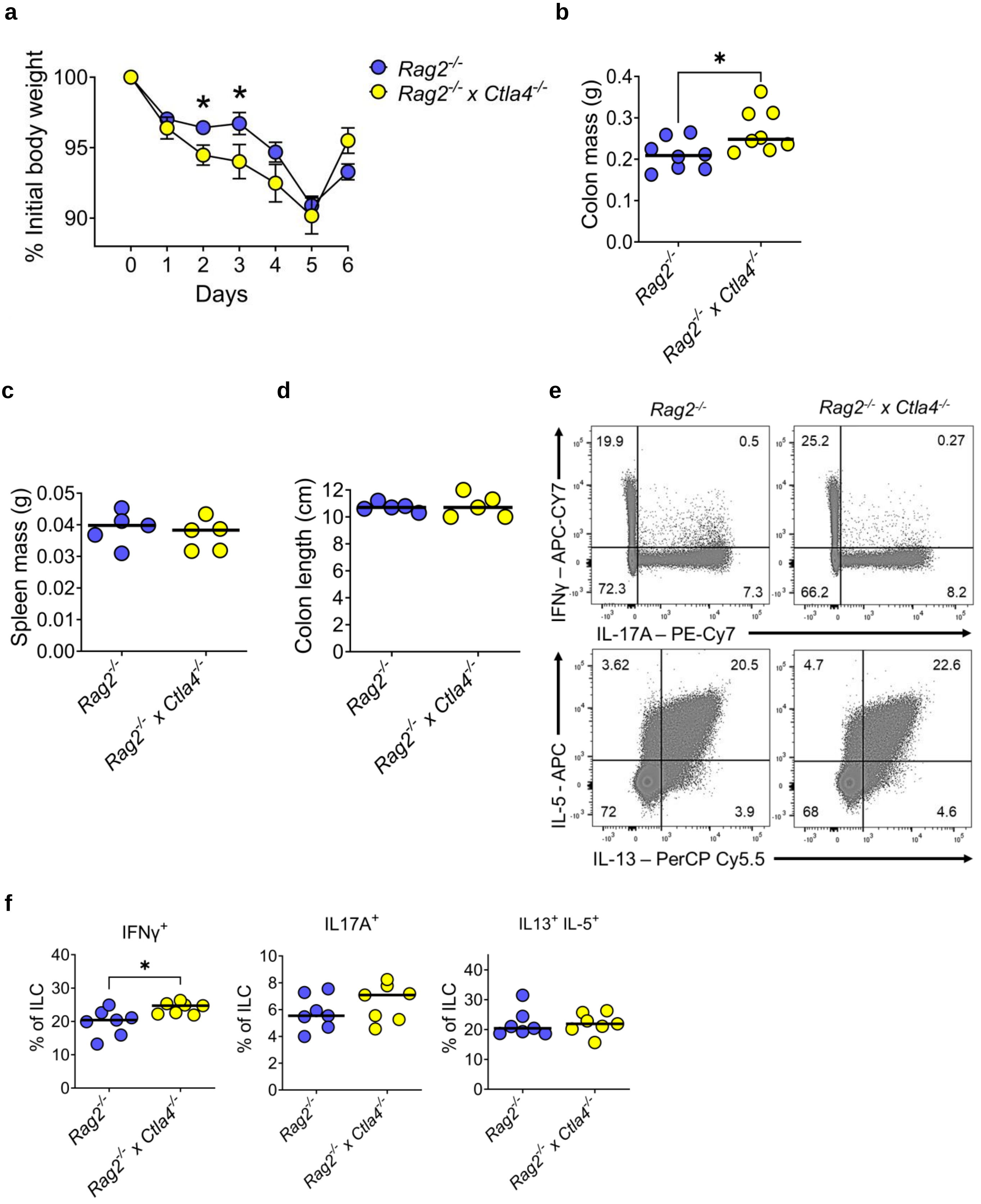

Supplementary Figure 15. DSS treated *$Rag2^{-/-}$ x $Ctla4^{-/-}$* mice suffer worse colitis
(a) Weight change, **(b)** colon mass, **(c)** spleen mass and **(d)** colon length of *$Rag2^{-/-}$ x $Ctla4^{-/-}$* mice (n=8, or n=5 for c and d) and *$Rag2^{-/-}$* mice (n=8, or n=5 for c and d) treated with 5% DSS. Colon mass, spleen mass and colon length measured on Day 2. **(e)** Representative flow cytometry plots and **(f)** summary dot plot showing IFNγ, IL17A and IL13+/IL5+ cytokine production from ILCs from the cLP in *$Rag2^{-/-}$ x $Ctla4^{-/-}$* mice (n=7) and *$Rag2^{-/-}$* mice (n=7) treated with 5% DSS. * $P<0.05$ Mann Whitney U test.

a

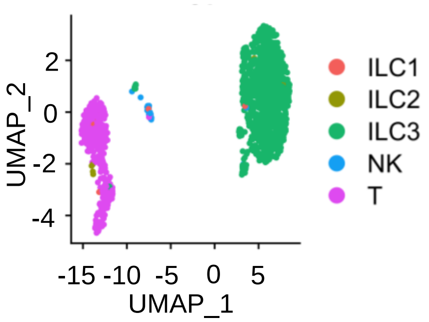

b

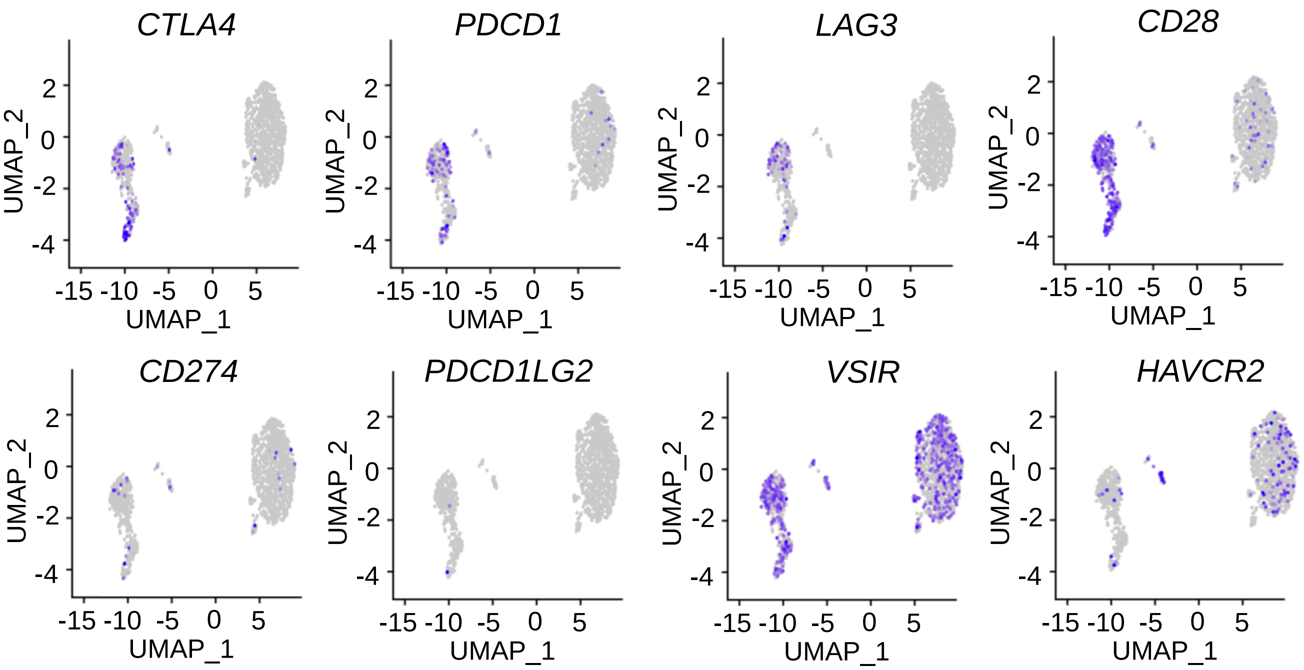

Supplementary Figure 16. The immune checkpoint transcriptional landscape across human intestinal ILC clusters

(a) UMAP plots and **(b)** violin plots showing the expression of different immune checkpoints across the three subsets of ILC, NK cells and T cells identified by Mazzurana et al.

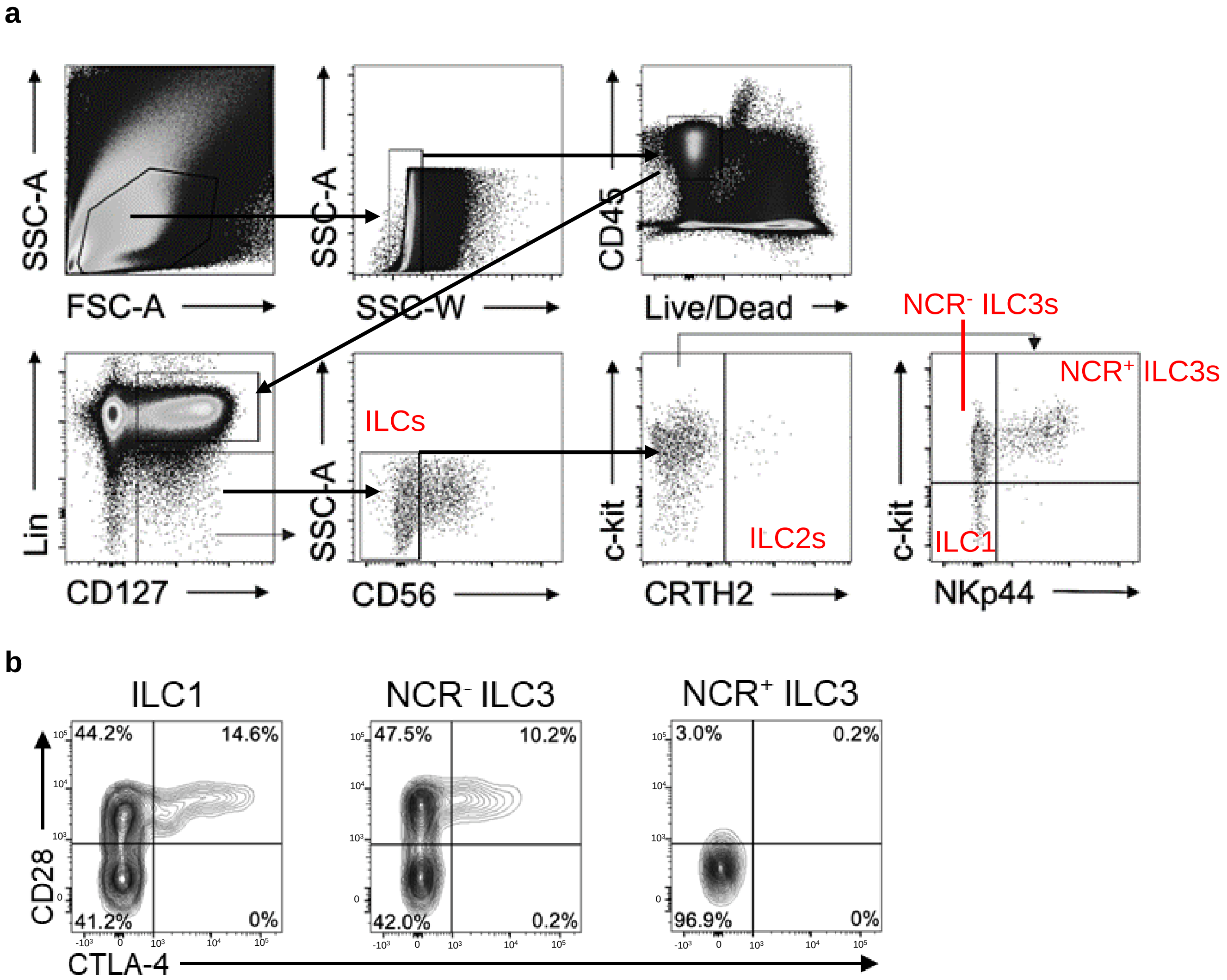

Supplementary Figure 17. Analysis of human ILC subsets in IBD patients
(a) Gating strategy used for identifying the different ILC subsets from the colonic lamina propria mononuclear cells from biopsies of IBD patients and healthy controls **(b)** Representative flow plot showing co-expression of CD28 and CTLA-4 on ILC1s, NCR^{-} ILC3s and NCR^{+} ILC3s

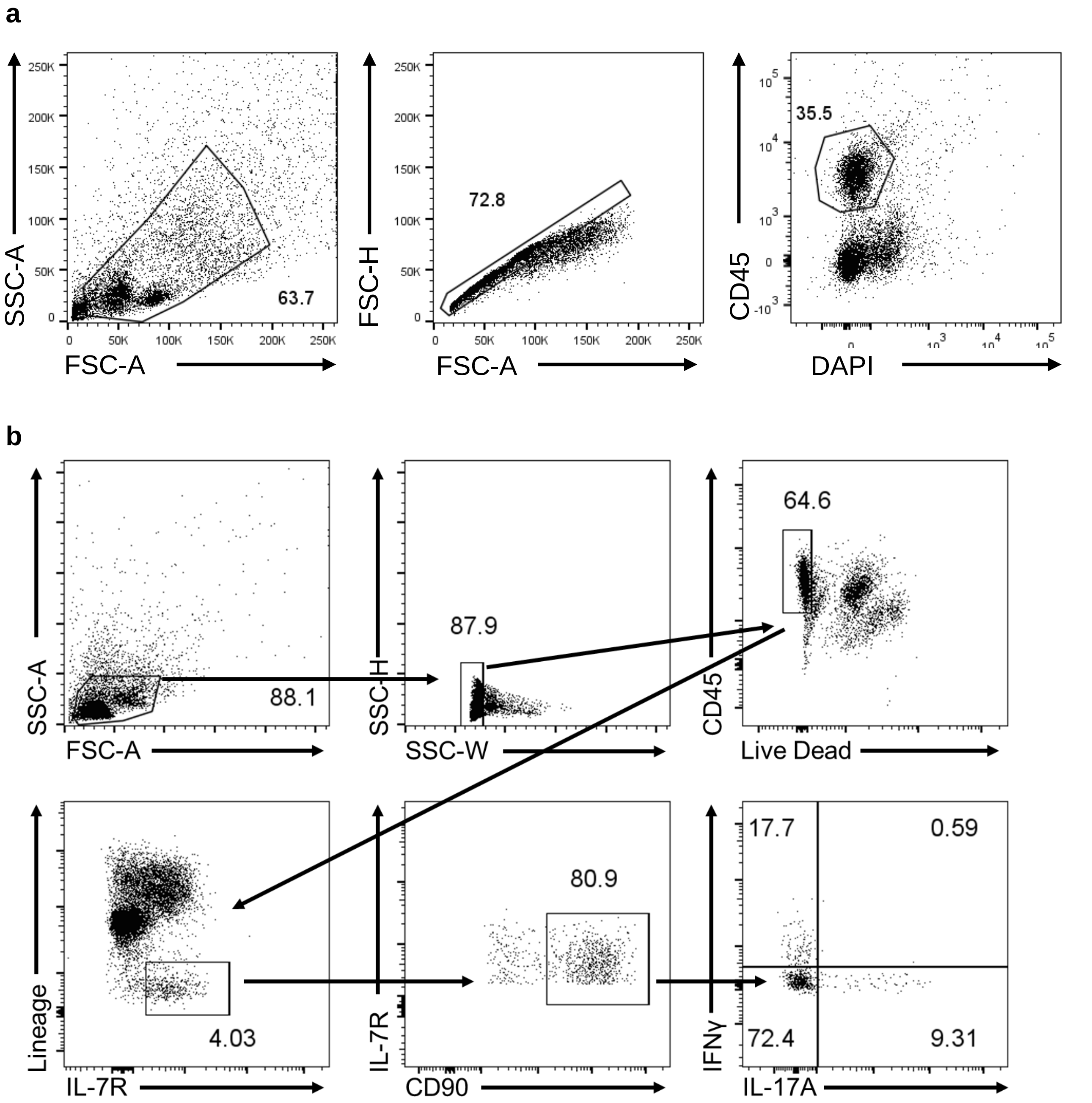

Supplementary Figure 18. Gating strategies for sorting and analysis of murine CPI-induced colitis
(a) Gating strategy used for sorting Live CD45 lymphocytes which were then immediately run on the 10X Genomics Chromium **(b)** Gating strategy used for identifying the different ILC subsets from the colonic lamina propria mononuclear cells from wild-type mice treated with CPI.

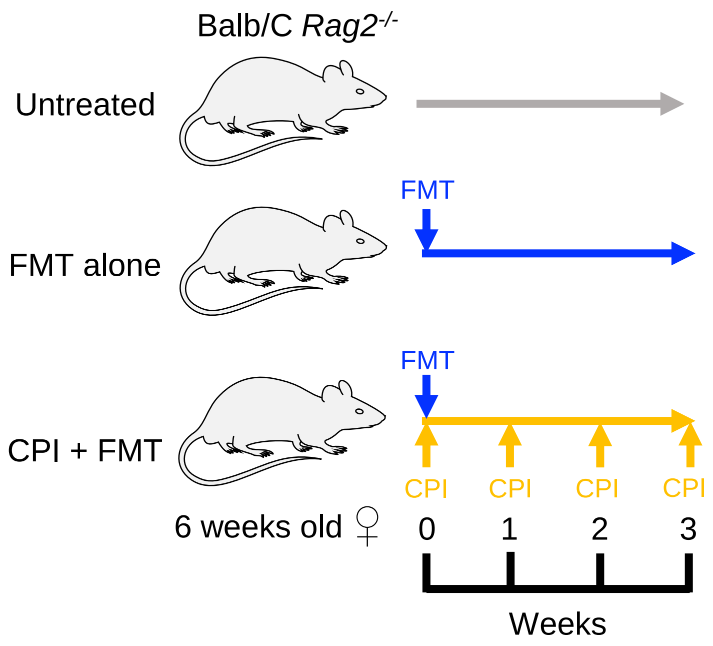

Supplementary Figure 19. Experimental plan for inducing CPI-colitis in *Rag2*$^{-/-}$ mice
Schematic of the experimental plan for the CPI-colitis in *Rag2*$^{-/-}$ mice.

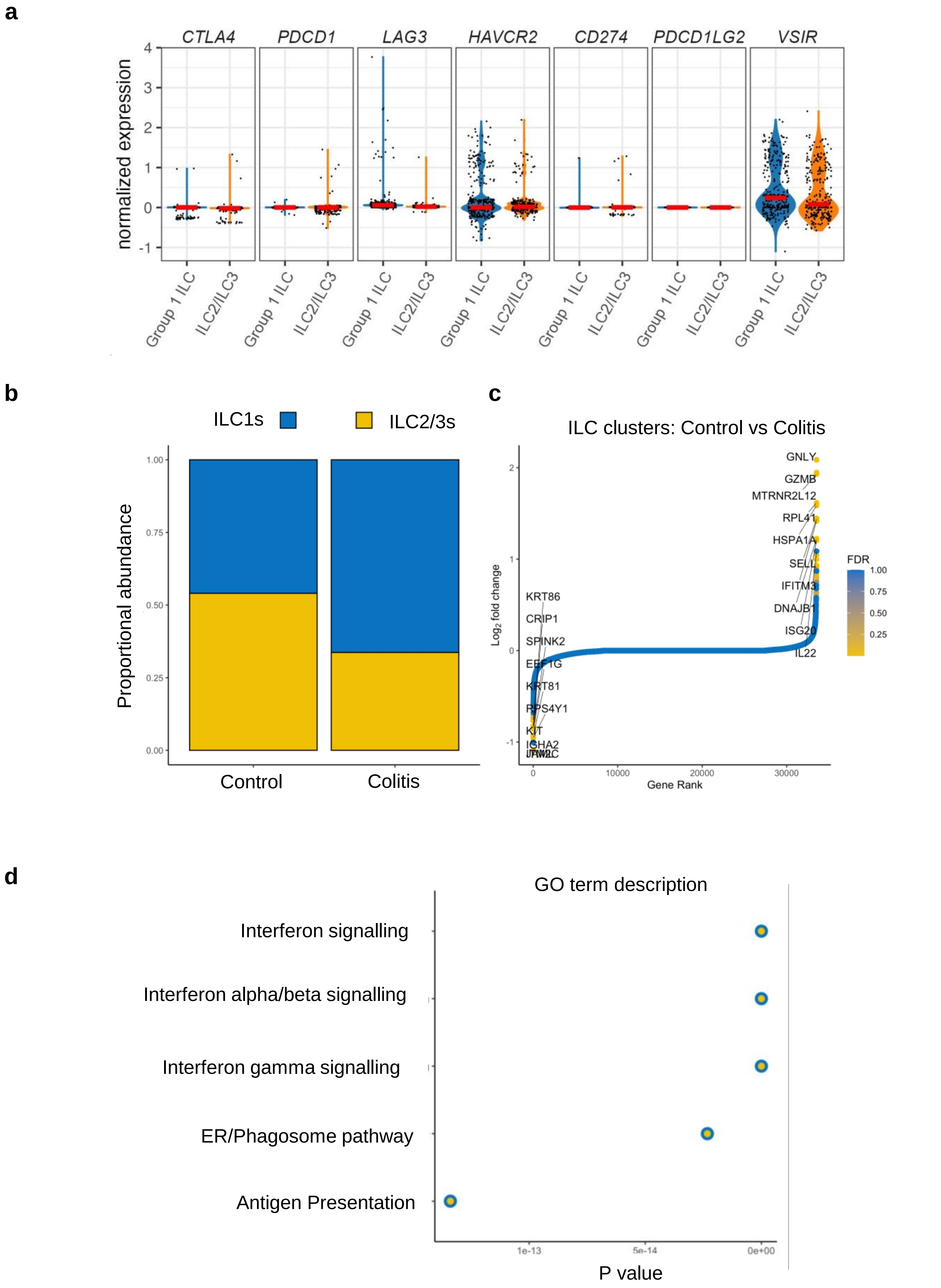

Supplementary Figure 20. ILC1 subsets are expanded in patients with CPI-colitis

(a) Violin plots of the normalised gene expression of typical immune checkpoint genes from healthy controls between the two ILC clusters identified by Luoma et al. **(b)** Cell cluster abundance proportion changes between the 2 ILC clusters identified by Luoma et al. **(c)** Chair plot showing differentially expressed genes in the ILC clusters ranked by increasing fold change. **(d)** Pathway analysis showing the most significantly increased GO pathways in the ILCs clusters.

Supplementary Table 1. List of flow cytometry antibodies used

Antibody	Clone	Species Reactivity	Fluorochrome	Company
Live Dead		Both	Fixable blue or aqua or 700	Thermo Fisher
CD45.2	104	Murine	BUV395	Biolegend
Hematopoietic Lineage Cocktail	consisting of: CD3 (17A2), B220 (RA3-6B2), CD11b (M1/70), TER-119 (TER-119), Gr-1 (RB6-8C5)	Murine	Fitc	Thermo Fisher
CD5	53-7.3	Murine	Fitc	Thermo Fisher
CD127	A7R34	Murine	APC	Biolegend
CD28	37.51	Murine	Biotin	Biolegend
Streptavidin			PE	BD
RORγt	Q31-378	Murine	BV785	BD
IL-10	JES5-16E3	Murine	PE	Thermo Fisher
IL-5	TRFK5	Murine	PE	Biolegend
IL-13	eBio13A	Murine	PerCP-ef710	Thermo Fisher
IFNγ	XMG1.2	Murine	BUV737	Biolegend / Thermo Fisher
IL-17A	eBio17B7	Murine	PE-Cy7	Thermo Fisher
Nkp46	29A1.4	Murine	BV605	Biolegend
CCR6	29-21.17	Murine	BV421	Biolegend
CTLA-4	UC10-4F10-11	Murine	PE-CF594	BD
KLRG1	2F1	Murine	APC-Cy7	Thermo Fisher
Gr-1	RB6-8C5	Murine	Fitc	Thermo Fisher
CD11b	M1/70	Murine	PE-Cy7	BD Biosciences
CD90.2	53-2.1	Murine	ef450	Thermo Fisher
ICOS	C398.4A	Murine	PE-Cy7	Thermo Fisher
ST2	RMST2-2	Murine	BUV737	Thermo Fisher
Hematopoietic Lineage Cocktail	consisting of: CD2 (RPA-2.10), CD3 (OKT3), CD19 (HIB19), CD14 (61D3), CD16 (CB16), CD56 (TULY56), CD235a (HIR2)	Human	Fitc	Thermo Fisher
CRTH2	BM16	Human	BV421	Biolegend
CD56	QA17A16	Human	Pacific Blue	Biolegend
c-kit	ACK2	Human	PE-Cy7	Thermo Fisher
NKp44	P44-8	Human	APC	Biolegend
CTLA-4	BN13	Human	PE	Biolegend
CD28	CD28.2	Human	PE-Dazzle 594	Biolegend
CD45	HI30	Human	Pacific Orange	Thermo Fisher
