# Supplementary material for: CTLA-4 expressing innate lymphoid cells modulate mucosal homeostasis in a microbiota dependent manner
Source: Nat Commun. 2024 Nov 4;15:9520. doi: 10.1038/s41467-024-51719-6 (PMC11535242; doi:10.1038/s41467-024-51719-6)
Supplement: Supplementary file 3 — Description of Additional Supplementary Files [file 41467_2024_51719_MOESM3_ESM.pdf]

Description of Additional Supplementary Files

Supplementary Data 1: Demographic patient meta data from consented volunteers in Figure 5a
