# Supplementary material for: CTLA-4 expressing innate lymphoid cells modulate mucosal homeostasis in a microbiota dependent manner
Source: Nat Commun. 2024 Nov 4;15:9520. doi: 10.1038/s41467-024-51719-6 (PMC11535242; doi:10.1038/s41467-024-51719-6)
Supplement: Supplementary file 5 — Reporting Summary [file 41467_2024_51719_MOESM5_ESM.pdf]

nature portfolio

Corresponding author(s): Professor Graham Lord and Professor Nick Powell

Last updated by author(s): Apr 12, 2024

Reporting Summary

Nature Portfolio wishes to improve the reproducibility of the work that we publish. This form provides structure for consistency and transparency in reporting. For further information on Nature Portfolio policies, see our Editorial Policies and the Editorial Policy Checklist.

Statistics

For all statistical analyses, confirm that the following items are present in the figure legend, table legend, main text, or Methods section.

n/a	Confirmed	
☐	☒	The exact sample size (*n*) for each experimental group/condition, given as a discrete number and unit of measurement
☐	☒	A statement on whether measurements were taken from distinct samples or whether the same sample was measured repeatedly
☐	☒	The statistical test(s) used AND whether they are one- or two-sided<br>*Only common tests should be described solely by name; describe more complex techniques in the Methods section.*
☐	☒	A description of all covariates tested
☐	☒	A description of any assumptions or corrections, such as tests of normality and adjustment for multiple comparisons
☐	☒	A full description of the statistical parameters including central tendency (e.g. means) or other basic estimates (e.g. regression coefficient) AND variation (e.g. standard deviation) or associated estimates of uncertainty (e.g. confidence intervals)
☐	☒	For null hypothesis testing, the test statistic (e.g. *F*, *t*, *r*) with confidence intervals, effect sizes, degrees of freedom and *P* value noted<br>*Give P values as exact values whenever suitable.*
☒	☐	For Bayesian analysis, information on the choice of priors and Markov chain Monte Carlo settings
☒	☐	For hierarchical and complex designs, identification of the appropriate level for tests and full reporting of outcomes
☒	☐	Estimates of effect sizes (e.g. Cohen's *d*, Pearson's *r*), indicating how they were calculated

Our web collection on statistics for biologists contains articles on many of the points above.

Software and code

Policy information about availability of computer code

Data collection	Reported in the Methods section of the manuscript.
Data analysis	Described in the Methods and Results sections of the manuscript.<br><br>NGS data presented in the manuscript have been made publicly available through the Gene Expression Omnibus (GEO) database found at https://www.ncbi.nlm.nih.gov/geo/. GSE208395 " Transcriptome profiling of colon biopsies from pre-clinical models of colitis" for the bulk RNA-seq comparing different pre-clinical mouse models used for Figure 4a (https://www.ncbi.nlm.nih.gov/geo/query/acc.cgi?acc=GSE208395). GSE254247 for Figure 4b-c comparing colons of Rag2-/- against Rag2-/- x Ctla4-/- titled "Transcriptomic profiling of the role of Ctla4 in the innate immune system" (https://www.ncbi.nlm.nih.gov/geo/query/acc.cgi?acc=GSE254247). GSE224758 for Figure 5a comparing healthy controls with patients with ulcerative colitis titled "Gene expression profiling of colon biopsies from ulcerative colitis patients and healthy volunteers" (https://www.ncbi.nlm.nih.gov/geo/query/acc.cgi?acc=GSE224758). Lastly, GSE222959 titled "Single cell transcriptomics reveals colonic lymphocyte remodelling and emergence of polyfunctional, cytolytic lymphocyte responses in CPI-induced colitis" for the single cell RNA-seq data comparing the wildtype CPI+FMT treated BALB/c CPI-C mice to healthy untreated wildtype BALB/c mice used in Figures 6 (https://www.ncbi.nlm.nih.gov/geo/query/acc.cgi?acc=GSE222959)

For manuscripts utilizing custom algorithms or software that are central to the research but not yet described in published literature, software must be made available to editors and reviewers. We strongly encourage code deposition in a community repository (e.g. GitHub). See the Nature Portfolio guidelines for submitting code & software for further information.

Data

Policy information about availability of data

All manuscripts must include a data availability statement. This statement should provide the following information, where applicable:

- Accession codes, unique identifiers, or web links for publicly available datasets
- A description of any restrictions on data availability
- For clinical datasets or third party data, please ensure that the statement adheres to our policy

NGS data presented in the manuscript have been made publicly available through the Gene Expression Omnibus (GEO) database found at https://www.ncbi.nlm.nih.gov/geo/. GSE208395 " Transcriptome profiling of colon biopsies from pre-clinical models of colitis" for the bulk RNA-seq comparing different pre-clinical mouse models used for Figure 4a (https://www.ncbi.nlm.nih.gov/geo/query/acc.cgi?acc=GSE208395). GSE254247 for Figure 4b-c comparing colons of Rag2-/- against Rag2-/- x Ctla4-/- titled "Transcriptomic profiling of the role of Ctla4 in the innate immune system" (https://www.ncbi.nlm.nih.gov/geo/query/acc.cgi?acc=GSE254247). GSE224758 for Figure 5a comparing healthy controls with patients with ulcerative colitis titled "Gene expression profiling of colon biopsies from ulcerative colitis patients and healthy volunteers" (https://www.ncbi.nlm.nih.gov/geo/query/acc.cgi?acc=GSE224758). Lastly, GSE222959 titled "Single cell transcriptomics reveals colonic lymphocyte remodelling and emergence of polyfunctional, cytolytic lymphocyte responses in CPI-induced colitis" for the single cell RNA-seq data comparing the wildtype CPI+FMT treated BALB/c CPI-C mice to healthy untreated wildtype BALB/c mice used in Figures 6 (https://www.ncbi.nlm.nih.gov/geo/query/acc.cgi?acc=GSE222959)

Research involving human participants, their data, or biological material

Policy information about studies with human participants or human data. See also policy information about sex, gender (identity/presentation), and sexual orientation and race, ethnicity and racism.

Reporting on sex and gender	Included in methods and supplementary Data 1. Meta-data was gathered where available, in brief, there were 6 female and 10 males with UC
Reporting on race, ethnicity, or other socially relevant groupings	Included in methods and supplementary Data 1. Meta-data was gathered where available, in brief, there were a mix of ethnicities but the majority were Caucasian.
Population characteristics	Included in methods and supplementary Data 1
Recruitment	Included in methods
Ethics oversight	Included in methods . Studies in human tissues received ethical approval from Guy's and St Thomas's Trust (REC number: 15/LO/1998).

Note that full information on the approval of the study protocol must also be provided in the manuscript.

Field-specific reporting

Please select the one below that is the best fit for your research. If you are not sure, read the appropriate sections before making your selection.

[x] Life sciences [ ] Behavioural & social sciences [ ] Ecological, evolutionary & environmental sciences

For a reference copy of the document with all sections, see nature.com/documents/nr-reporting-summary-flat.pdf

Life sciences study design

All studies must disclose on these points even when the disclosure is negative.

Sample size	Sample sizes were determined per experiment. For these animal experiments, in general experiments had around 3 or 4 of each condition per experiment and then repeated at least twice when possible. For human samples, a n of 5 was used for healthy control and IBD. Statistical analyses between 2 groups will include t-tests and ANOVA (parametric data) and Mann-Whitney U-tests and Kruskal-Wallis tests (non-parametric data).
Data exclusions	No data has been excluded
Replication	Most experiments were repeated and performed independently. All attempts shown were successful and included in the Source Data
Randomization	Mice were randomised in the cage and to ensure no cage variation etc CPI+FMT were mixed in the same cage as FMT only, or Rag-/- control and Rag-/- receiving anti-CD40 in the same cage, as examples. Only for microbiome related experiments were mice kept separate as control untreated mice, due to the FMT and not wanting to alter the control mice microbiome within the cage environment or having no . RNA samples were randomly chosen out of the main cohort of experiments as long as they passed QC, but sequenced at the same time to minimise batch effects.
Blinding	Investigators (bioinformaticians etc) were blinded to the condition of each mouse by being given generic numbers for the mouse sample e.g. listing the mice as WT1-16 instead of by condition when they were analysing the transcriptomics.

Reporting for specific materials, systems and methods

We require information from authors about some types of materials, experimental systems and methods used in many studies. Here, indicate whether each material, system or method listed is relevant to your study. If you are not sure if a list item applies to your research, read the appropriate section before selecting a response.

Materials & experimental systems

n/a	Involved in the study
☐	☒ Antibodies
☒	☐ Eukaryotic cell lines
☒	☐ Palaeontology and archaeology
☐	☒ Animals and other organisms
☒	☐ Clinical data
☒	☐ Dual use research of concern
☒	☐ Plants

Methods

n/a	Involved in the study
☒	☐ ChIP-seq
☐	☒ Flow cytometry
☒	☐ MRI-based neuroimaging

Antibodies

Antibodies used	anti-CTLA-4 (9H10, BioXCell), anti-PD-1 (RMP1-14, BioXCell), anti-CD40 (FGK4.5, BioXCell). Control-isotype clones used were 2A3 (rat IgG2a) and HRPN (rat IgG1)
Validation	Antibodies were used from experience on previous experiments, (Wei SC, et al. Distinct Cellular Mechanisms Underlie Anti-CTLA-4 and Anti-PD-1 Checkpoint Blockade. Cell 170, 1120-1133.e1117 (2017), Powell N, et al. The transcription factor T-bet regulates intestinal inflammation mediated by interleukin-7 receptor+ innate lymphoid cells. Immunity 37, 674-684 (2012) and Lo et al.Immune checkpoint inhibitor-induced colitis is mediated by polyfunctional lymphocytes and is dependent on an IL23/IFNγ axis. Nature communications, and published data by the company website

Animals and other research organisms

Policy information about studies involving animals; ARRIVE guidelines recommended for reporting animal research, and Sex and Gender in Research

Laboratory animals	C57BL/6 and BALB/c wild-type mice (both Charles River) and BALB/c Rag2-/- mice and BALB/c Cd28-/- mice (Taconic) were sourced commercially. A colony of colitis-free Rag2-/- x Tbx21-/- (TRnUC) mice was generated as described previously. BALB/c Ctla4-/- mice were kindly provided by A. Sharpe (Harvard, Boston) and used to generate Rag2-/- x Ctla4-/- mice. RorcGFP mice were a kind gift of Dr Gérard Eberl. BALB/c Rag2-/- mice and BALB/c Cd28-/- mice were crossed to generate the Rag2-/- x Cd28-/- mice. All these mice were housed in specific pathogen–free facilities at King's College London Biological Services Unit, Imperial College London Central Biomedical Services, University College London Biological Services Unit or Charles River Laboratories. C57BL/6 germ-free mice were housed in germ-free facilities at St. George's University London and also at the University of Manchester. Breeding of Rag2-/- x Ctla4-/- mice, Rag2-/- x Cd28-/- mice and Rag2-/- mice was performed at University College London under Home Office Licences PPL PA8A94052 and PP5389651. These mice were housed in individually ventilated cages in a temperature- and humidity-controlled environment with a 14-h light and 10-h dark cycle and ad libitum feeding. Animals were provided with environmental enrichment including cardboard tunnels, paper houses, chewing blocks and aspen wood wool nesting material.
Wild animals	This study did not involve wild mice
Reporting on sex	No sex biased was used here
Field-collected samples	This study does not include any field collected samples
Ethics oversight	All procedures were conducted under licenses (Home Office Licence Numbers PPL: 70/6792, 70/8127, 70/7869, P8999BD42 PA8A94052, PP5389651) from the United Kingdom (UK) Home Office in accordance with The Animals (Scientific Procedures) Act 1986 and licences were approved by each Animal Welfare and Ethical Review Body.

Note that full information on the approval of the study protocol must also be provided in the manuscript.

Plants

Seed stocks	N/A
Novel plant genotypes	N/A
Authentication	N/A

Flow Cytometry

Plots

Confirm that:

- [x] The axis labels state the marker and fluorochrome used (e.g. CD4-FITC).
- [x] The axis scales are clearly visible. Include numbers along axes only for bottom left plot of group (a 'group' is an analysis of identical markers).
- [x] All plots are contour plots with outliers or pseudocolor plots.
- [x] A numerical value for number of cells or percentage (with statistics) is provided.

Methodology

Sample preparation	Mouse and human acolons were excised and placed in cold Phosphate Buffered Saline (PBS) solution. The tissue was digested in HBSS with 2% FCS and supplemented with 0.5mg/ml collagenase D, 10 g/ml DNase I and 1.5mg/ml dispase II (all Roche). The digested lymphocyte-enriched population was harvested using a 40%-80% Percoll gradient centrifugation for cLP.<br><br>Single suspension extracted cells, as described above, were plated into flow cytometry tubes (Sarstedt) at a concentration of 1 x 10^6 per ml. Cells were stimulated with 50ng/ml phorbol 12-myristate 13-acetate (PMA), 1 g/ml ionomycin, 2M monensin (all Sigma Aldrich) for 3-4 hours. FcR receptor blocking antibodies were added before staining with antibodies.<br>Surface staining antibodies were added with live/dead stain (Invitrogen). For intracellular staining, cells were fixed and permeabilised using the Foxp3 fixation/permeabilization buffer kit (Thermo Fisher) according to the manufacturer's instructions.
Instrument	Samples were acquired using a BD LSRFortessa or a BD Symphony (BD Biosciences).
Software	Sample data was recorded in FCS 3.0 data format using BD FACSDiva 6.0 software (BD Biosciences). Analysis of the data was performed using FlowJo software (Treestar Inc., Ashland, OR, USA).
Cell population abundance	Cell populations were sorted using a BD Aria. Purity were measured with an automated cell counter to judge cell count and viability.
Gating strategy	Gating strategies are included in the Supplementary Figures

- [x] Tick this box to confirm that a figure exemplifying the gating strategy is provided in the Supplementary Information.
